# In vivo microsurgical training with the symani^®^ robot: early experience and feasibility

**DOI:** 10.1007/s11701-026-03506-2

**Published:** 2026-07-20

**Authors:** Alessandro Piperno, Enrico Palombo, Maria Francesca Pennarola, Simone Otera, Sigismondo Castaldo, Pierluigi Tos, Alessia Pagnotta

**Affiliations:** 1 Hand and Microsurgery Unit, Jewish Hospital of Rome, Rome, Italy; 2 Department of Anatomical, Forensic Medicine and Orthopaedic Sciences, Sapienza University of Rome, Histological, Rome, Italy; 3 U.O.C. Ricerca, Formazione & Cooperazione Internazionale - A.O.R.N.A. Cardarelli, Naples, Italy; 4 Hand Surgery and Reconstructive Microsurgery Department, ASST Centro Specialistico Ortopedico Traumatologico Gaetano Pini-CTO, Milan, Italy; 5 Department of Surgery, Plastic Surgery Unit, Sapienza University of Rome, Rome, Italy

**Keywords:** In vivo microsurgical training, Robotic surgery, Symani^®^, Hand microsurgery, Reconstructive microsurgery

## Abstract

Robotic microsurgery offers important advantages in vascular anastomoses and nerve sutures, particularly for structures < 1 mm as required in supermicrosurgery. The Symani^®^ surgical robot provides micrometric movements and tremor elimination through motion scaling, improving precision and reducing operator fatigue. Although several studies have investigated Symani-related training and learning curves, most were conducted on synthetic or in vitro animal models. The few studies performed on in vivo rat models involve microvascular surgeons with many years of experience in traditional microsurgery, rather than residents with no experience in robotic microsurgery and only limited exposure to conventional microsurgery. During the Advanced Microsurgery Course 2025 in Naples (Italy), held between May and December 2025, 14 participants with no prior robotic microsurgery experience and minimal conventional microsurgical training performed first a practice session on a silicone synthetic model to become familiar with the robot, and subsequently on in vivo murine models using the Symani^®^ robot. Data from the in vivo exercise were collected and compared, focusing on time required to complete individual knots and intraoperative complications. Participants demonstrated rapid acquisition of operative skills, showing a reduction in suturing time during in vivo procedures, which indicates a fast adaptation to the robotic platform even among inexperienced operators. The Symani^®^ robotic microsurgery system is intuitive and associated with a steep learning curve, even for users with no prior exposure to robotic microsurgery and only basic training in traditional microsurgery. This supports its potential as an effective training platform and clinical tool in supermicrosurgery.

## Introduction

The development of robotic microsurgery has enabled increasingly precise vascular anastomoses and nerve sutures, especially in small-caliber vessels < 1 mm, which fall within the field of supermicrosurgery; however, supermicrosurgery was already possible long before the advent of robotics, with highly precise hand-sewn anastomoses achieving good outcomes. In general terms, supermicrosurgery may be defined as a microsurgical technique focused on vascular and nerve anastomoses in vessels ≤ 1 mm in diameter, typically performed under high magnification, enabling the reconstruction of extremely small-caliber structures with high precision. Robotic systems, such as the Symani surgical robot, represent a valuable advancement that may further enhance precision, stability, and reproducibility, as well as reduce surgeon fatigue. The system consists of:

- a mobile cart equipped with robotic arms (macro-positioners),
- Nanowrist instruments, available for both microsurgical and supermicrosurgical applications.
- a control unit with an electromagnetic field within which the surgeon moves the joysticks.

The joystick movements are detected with high precision and translated into corresponding movements of the Nanowrist instruments. As with any advanced robotic system, the Symani^®^ robot requires a dedicated learning curve and structured training, not only for the operating surgeon but also for the entire surgical team, in order to ensure optimal integration and correct implementation of the device within the operating room [1]. To date, several studies have assessed the learning curve of both experienced and less experienced surgeons. Some of these studies were conducted on synthetic models using silicone tubes, others on in-vitro animal models, and still others on in-vivo models [2–7]. In all cases, however, the participants already had some degree of microsurgical training. The aim of our study is to evaluate the in vivo application of the Symani^®^ robot in operators with limited experience in conventional microsurgery and no prior exposure to robotic microsurgery. This study was designed as an exploratory, descriptive feasibility study aimed at characterizing early learning patterns in novice users.

## Methods

The study was conducted during an advanced Microsurgery course held in Naples between May and December 2025, involving 14 physicians with no prior experience in robotic microsurgery and only minimal experience in traditional microsurgery [8]. The primary objective was to evaluate the trend in the time required to perform individual suturing stitches in novice participants while simultaneously monitoring the most frequent technical errors. The course was primarily designed as a training program in conventional manual microsurgery, which represents the foundational skill set for all participants and constitutes the main objective of the program. Accordingly, in vivo training on a rat model, properly anesthetized and sedated by a certified anesthesiologist, was first used to allow participants to practice standard manual microsurgical vascular anastomoses. In a subsequent phase, and in preparation for robotic-assisted procedures, participants trained on a synthetic model consisting of a 2-mm silicone tube, focusing on the use of the robotic platform in a simulated environment. Finally, robotic-assisted vascular anastomoses were performed on the same rat previously used for manual microsurgical training, thereby avoiding the use of additional animals and limiting potential ethical concerns associated with exposing a new animal model to participants without prior experience in robotic surgery.

**Fig. 1:**
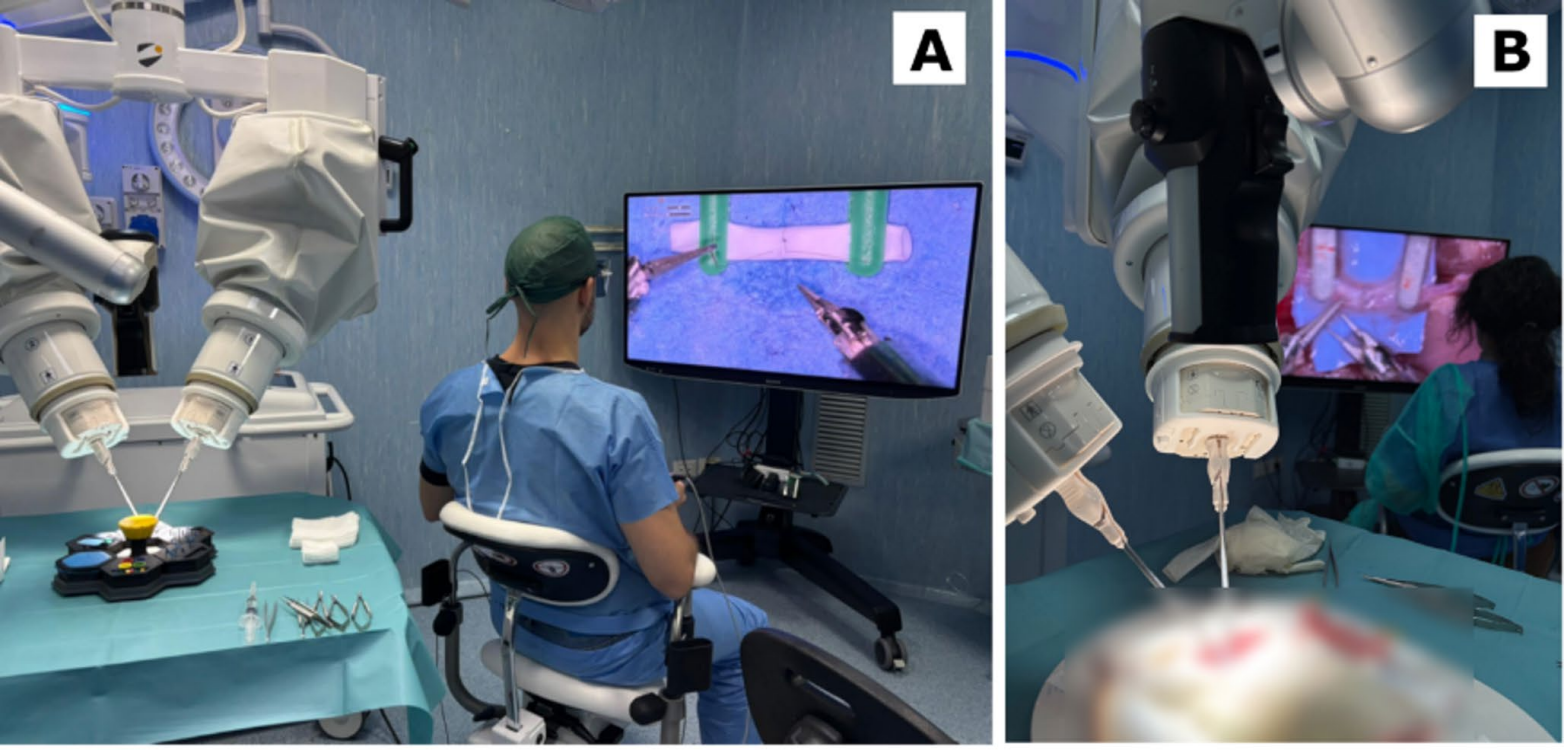
**A**) Training session with the Symani^®^ microsurgical robot in exoscope configuration on a 2-mm-⌀ silicone tube. **B**) End-to-end anastomosis performed on the femoral artery in an in vivo rat model

Participants were first required to perform a single practice stitch on the synthetic silicone model as a brief familiarization step. They subsequently proceeded directly to the in vivo model, where they were instructed to place as many suturing stitches as possible within a time frame of approximately 15 min, under the supervision of the robotic system representative, who provided instructional guidance on the correct use of the device and verbal support in case of technical or procedural difficulties. However, in some instances, the effective available operative time varied among participants, as part of the allocated session was occasionally dedicated to additional verbal explanations of the procedural steps by the system representative, which reduced the time effectively spent on hands-on execution. The in vivo procedure was performed on the femoral artery, which had an average diameter of approximately 1.0 mm, as measured intraoperatively using a caliper. All procedures were performed using the Symani^®^ robotic microsurgical system (MMI, Medical Microinstruments) in exoscope mode, with visualization provided by the Olympus Orbeye^®^ 4 K 3D digital microscope (Fig. 1).

The instruments employed were the NanoWrist^®^ robotic devices, specifically a dilator and a needle holder with 10 − 0 sutures. The motion-scaling reduction factor used during all procedures ranged between 7× and 10×. The timer was started when the participant began the first needle pass, with the needle already mounted on the robot’s needle holder and stopped upon completion of the third closing knot. During each session, the total execution time and any technical complications (such as suture breakage, vessel tearing, or difficulty in handling vessel edges) were recorded and used as variables to assess performance improvement, defined as a reduction in the time required to perform each individual suture and the absence of complications (Table 1). Due to time constraints, it was not possible to complete a full circumferential anastomosis; therefore, patency testing could not be performed, representing a limitation in the qualitative assessment of the anastomosis.

**Table 1:** Overview of variables included in the analysis, measurement methods, and their role in data interpretation

Variable	Measurement Method	Role in Analysis
**Time to complete task**	Recorded in minutes for each anastomosis	Quantitative performance metric used to evaluate procedural efficiency
**Vessel diameter**	Measured intraoperatively using a caliper	Standardization parameter influencing procedural complexity
**Suture rupture**	Direct intraoperative observation	Surrogate marker of major technical error and indicator of technical failure

The table summarizes the performance metrics recorded during the study, including operative time, vessel type, vessel diameter, and suture rupture events. For each variable, the method of measurement and its role in the overall analysis are specified to improve transparency and methodological clarity.

## Results

A total of 9 participants completed the training session on the in vivo rat model, performing a variable number of stitches on the femoral artery. The distribution of execution times was highly heterogeneous, reflecting differences in individual learning progression (Table 2).

**Table 2:** Individual suture times on the in vivo rat model. During the training session, three cases of suture thread breakage were recorded. The tables also report the mean values with standard deviation for each participant and mean execution times per single stitch between participants, showing progressive time reduction

Rat model	Vessel type	Vessel ⌀	Number of stitches	Stitch 1	Stitch 2	Stitch 3	Stitch 4	Stitch rupture		Mean execution time per participant ± sample SD (min)
								Yes	No	
Participant #1	Femoral artery	0,9 mm	5	05:35	03:50	04:29	03:25	X		4:20 ± 0:57
Participant #2	Femoral artery	1 mm	2	04:02	02:13				X	3:08 ± 1:17
Participant #3	Femoral artery	1,1 mm	2	06:54	04:38				X	5:46 ± 1:36
Participant #4	Femoral artery	1 mm	3	06:10	03:44	01:58		X		3:57 ± 2:07
Participant #5	Femoral artery	1,2 mm	4	02:32	04:15	03:34	03:04		X	3:21 ± 0:44
Participant #6	Femoral artery	1,1 mm	2	03:50	06:33				X	5:12 ± 1:56
Participant #7	Femoral artery	1,1 mm	4	03:10	02:49	04:01	03:00	X		3:15 ± 0:32
Participant #8	Femoral artery	1 mm	2	06:50	03:42				X	5:16 ± 2:13
Participant #9	Femoral artery	1 mm	3	05:00	03:18	03:02			X	3:47 ± 1:02
Mean execution times per stitch				04:54	03:54	03:25	03:10			

**Fig. 2:**
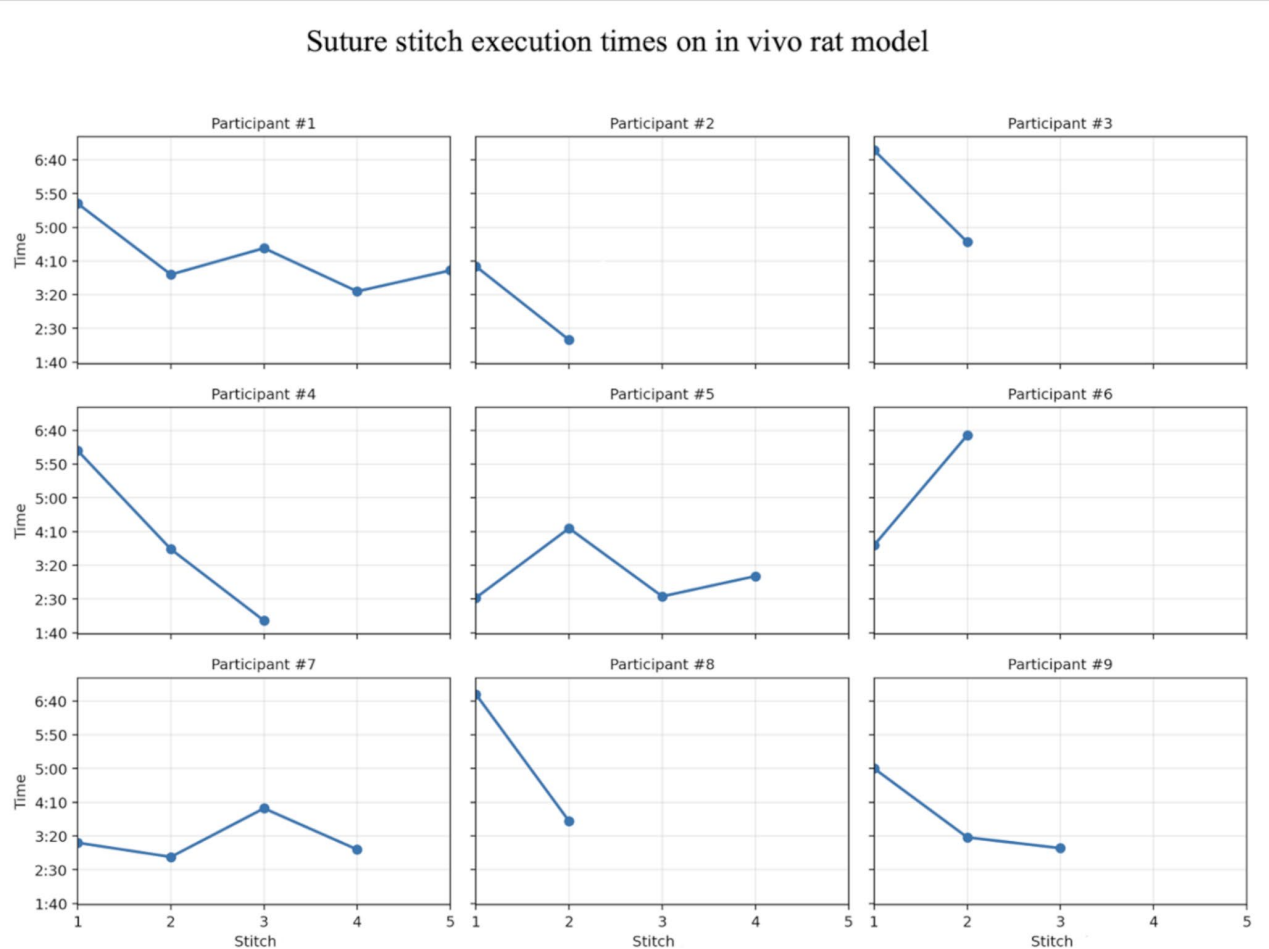
Individual suture times for each participant during in vivo training on the rat model, shown as a small multiples chart

To visualize these trends, a small multiples chart was generated, allowing direct comparison of each participant’s temporal evolution across successive stitches (Fig. 2).

A general trend toward reduced execution times was observed in most participants as they progressed through an increasing number of stitches. To further characterize performance variability, the sample standard deviation was calculated for each participant. The analysis showed heterogeneous patterns of performance consistency:

- Low variability (stable performance): Participants #7 and #5 (± 32 s and ± 44 s), indicating consistent and reproducible execution times.
- High variability with improvement trend: Participants #8 and #4 (SD > 1.5–2 min), showing progressive reductions in execution time, suggesting variability driven by learning.
- High variability with worsening trend: Participant #6, exhibiting a progressive increase in execution time, possibly related to fatigue or adaptation difficulties.

Overall, despite a general decrease in mean execution time across the cohort (learning effect), variability patterns indicate differences in performance stabilization and non-uniform skill acquisition. A Wilcoxon signed-rank test comparing consecutive suturing attempts demonstrated a reduction in execution time across subsequent stitches (*p* = 0.036), consistent with a learning curve effect. Technical adverse events were uncommon. No major complications such as vessel tearing occurred, although suture breakage was observed in 3 participants during their initial attempts on the rat model. These events did not prevent task completion and were successfully managed under supervision of the robotic system representative. The overall mean execution time decreased progressively from the earliest to the latest stitches, confirming a measurable learning effect even within a limited number of repetitions.

## Discussion

The findings of this study shows that novice operators with minimal prior exposure to traditional microsurgery and no experience in robotic microsurgery can rapidly acquire basic microsurgical skills using the Symani^®^ robotic system. Although most previously published learning-curve studies on robotic microsurgery have been conducted on experienced microsurgeons or trainees with at least some background in manual techniques, our cohort consisted entirely of beginners. The observation that absolute novices were able to progressively reduce their execution times across successive stitches suggests that the Symani^®^ system is intuitive and facilitates early technical improvement even in the absence of prior microsurgical experience. The analysis of the in vivo data revealed a downward trend in operative times for the majority of participants. Several participants demonstrated a predictable pattern, with the second or third stitch requiring substantially less time than the first, followed by stabilization around a personal performance plateau. Others showed more irregular learning curves, likely reflecting expected variability in early skill acquisition rather than reduced effectiveness of the Symani^®^ system. While not all participants exhibited a consistent reduction in execution time, the majority showed a downward trend, even if not strictly linear, in line with known patterns of novice fine motor skill learning. Nevertheless, the overall mean execution time declined progressively from the initial to the final stitch, indicating that even a limited number of repetitions was sufficient to trigger measurable improvement. More specifically, in our cohort, the mean execution time decreased from 4:54 for the first stitch to 3:10 for the fourth stitch, corresponding to an overall reduction of approximately 35%. This trend is comparable with the findings of Struebing et al. [2], who reported progressive improvement in robotic microsurgical task performance among novice users after repeated attempts on synthetic models. Similarly, Ballestín et al. [3] demonstrated that robotic assistance allowed stable microsurgical performance with reduced tremor and progressive reduction in execution time during repeated anastomotic tasks. Consistent with surgical learning curve theory, the progressive reduction in execution time observed after only a few repetitions reflects the early associative phase of motor skill acquisition, suggesting that robotic microsurgery training could be integrated early into microsurgical curricula rather than being reserved exclusively for experienced microsurgeons. Technical adverse events were infrequent, and their occurrence was compatible with early learning stages. Suture breakage, recorded in three participants, represents a common issue in microsurgical training and is typically associated with excessive traction or suboptimal needle handling. In this context, it is important to note that the Symani^®^ system does not provide haptic feedback: while this limitation is almost negligible when using extremely fine sutures such as 11/0, it may become more relevant with larger-caliber sutures like those used by our participants. In ultra-fine sutures such as 11/0, tactile perception is already extremely limited even during conventional manual microsurgery, and surgeons rely predominantly on visual cues to regulate traction forces. Conversely, with larger-caliber sutures such as 8/0–10/0, the absence of haptic feedback may become more relevant, particularly for novice operators, because tactile information normally contributes to tension modulation during needle passage and knot tightening. This may partially explain the occurrence of suture rupture during the early learning phase in our cohort [9]. As noted in previous studies, silicone-based models provide a useful preliminary platform, but they cannot reproduce the variability, fragility, and dynamic behavior of living vessels. For this reason, the rapid progression observed on the rat model is particularly significant. Our findings are consistent with previous reports describing a relatively steep learning curve for the Symani^®^ system [2, 3], even among operators with limited experience. Studies involving trained microsurgeons have emphasized the benefits of motion scaling, tremor filtration, and improved ergonomics, all of which reduce cognitive load and enhance microsurgical precision. The present work extends these observations indicating that these advantages may also facilitate early skill acquisition in complete beginners, that these advantages also facilitate early skill acquisition in complete beginners, supporting the use of robotic microsurgery as an effective teaching tool within structured training curricula. This study has several limitations. First, the number of stitches performed by each participant was not fixed but depended on the available operative time and individual execution speed, resulting in variable repetitions. This variability was inherent to the training setting and considered acceptable within the exploratory nature of the study. Second, a major limitation of this study is the lack of qualitative outcome measures, such as anastomotic patency or leakage. Due to time constraints, participants were unable to complete full circumferential anastomoses, preventing the use of validated quality assessment metrics. Consequently, execution time was adopted as a surrogate marker of early technical performance; however, it does not fully reflect procedural quality, and reductions in time should be interpreted primarily as increased familiarity with the robotic system rather than true surgical proficiency. Third, the study is limited by the small sample size, which reduces statistical power and limits the generalizability of the findings. Despite these limitations, the descriptive approach and visualization tools employed provide meaningful insight into the early learning dynamics associated with robotic microsurgery. Overall, this study demonstrates that the Symani^®^ robotic microsurgery system can be safely and effectively integrated into formal microsurgical training programs. Even participants with no prior microsurgical background were able to acquire foundational skills and demonstrate rapid improvement during in vivo procedures. Future studies incorporating standardized vascular targets, larger cohorts, and comparisons with experienced operators will be essential to further clarify the learning curve and define objective benchmarks for competency.

## Conclusions

The introduction of the Symani^®^ robot into microsurgery and supermicrosurgery may provide substantial support to the surgeon, as the system is intuitive and associated with a steep learning curve, even for users with no prior exposure to robotic microsurgery and only basic training in traditional microsurgery, supporting its potential as an effective training platform and clinical tool in supermicrosurgery. While most studies in the literature have focused on surgeons with prior experience [3–7], without exploring the system’s potential in completely novice operators, our feasibility study aims to evaluate the in vivo use of the Symani^®^ robot in personnel with limited experience in traditional microsurgery and no prior exposure to robotic microsurgery. This assessment is conducted within a structured training program and, importantly, on an in vivo animal model previously used for traditional microsurgery training and only subsequently, on the same animal, for robotic microsurgery. This approach was also adopted for ethical reasons, to minimize the number of animals used. The use of an in vivo model was intended to provide a more technically challenging environment for performing anastomoses. The data suggest that even beginners can rapidly acquire basic skills, performing the first stitches effectively and progressively improving anastomosis times over successive attempts. Moreover, the use of the robot by inexperienced operators may offer an advantage compared with experienced microsurgeons who have consolidated manual techniques and would need to adapt deeply ingrained motor skills to a different instrumental interface; overall, these preliminary observations support the efficiency of the Symani^®^ system as a training platform and highlight its potential role in promoting the development and dissemination of robotic micro and supermicrosurgery, with promising implications for both education and clinical practice. However, larger and standardized studies are necessary to confirm these preliminary findings and to better define the role of robotic systems in microsurgical training and clinical practice.

## Data Availability

No datasets were generated or analysed during the current study.
